# Untargeted Metabolomics Reveals Intervention Effects of Total Turmeric Extract in a Rat Model of Nonalcoholic Fatty Liver Disease

**DOI:** 10.1155/2016/8495953

**Published:** 2016-05-30

**Authors:** Ya Wang, Ming Niu, Ge-liu-chang Jia, Rui-sheng Li, Ya-ming Zhang, Cong-en Zhang, Ya-kun Meng, He-rong Cui, Zhi-jie Ma, Dong-hui Li, Jia-bo Wang, Xiao-he Xiao

**Affiliations:** ^1^Pharmacy College, Jinzhou Medical University, Jinzhou 121000, China; ^2^China Military Institute of Chinese Medicine, 302 Military Hospital, Beijing 100039, China; ^3^Animal Laboratory Center, 302 Hospital of PLA, Beijing 100039, China; ^4^Beijing Friendship Hospital, Capital Medical University, Beijing 100050, China

## Abstract

Nonalcoholic fatty liver disease (NAFLD) is one of the most common forms of chronic liver disease. Currently, there are no recognized medical therapies effective for NAFLD. Previous studies have demonstrated the effects of total turmeric extract on rats with NAFLD induced by high-fat diet. In this study, serum metabolomics was employed using UHPLC-Q-TOF-MS to elucidate the underlying mechanisms of HFD-induced NAFLD and the therapeutic effects of TE. Supervised orthogonal partial least-squares-discriminant analysis was used to discover differentiating metabolites, and pathway enrichment analysis suggested that TE had powerful combined effects of regulating lipid metabolism by affecting glycerophospholipid metabolism, glycerolipid metabolism, and steroid hormone biosynthesis signaling pathways. In addition, the significant changes in glycerophospholipid metabolism proteins also indicated that glycerophospholipid metabolism might be involved in the therapeutic effect of TE on NAFLD. Our findings not only supply systematic insight into the mechanisms of NAFLD but also provide a theoretical basis for the prevention or treatment of NAFLD.

## 1. Introduction

As a chronic disease, nonalcoholic fatty liver disease is acknowledged to be the hepatic manifestation of obesity and metabolic syndrome [1], presenting an increasing incidence worldwide. NAFLD severity encompasses a wide spectrum, ranging from simple steatosis to more severe nonalcoholic steatohepatitis, involving inflammation and apoptosis with or without fibrosis and cirrhosis. To date, conventional and modern drugs used to treat NAFLD are sometimes insufficient and can have serious side effects [2, 3]. Therefore, there is no effective and safe medical therapy available for NAFLD. Continuous effort to develop a promising pharmacological therapy for the treatment of NAFLD is still urgently needed.

Traditional Chinese medicine has been practiced in China for centuries and its application in the prevention of a variety of chronic diseases [4, 5]. The perennial herb Curcuma longa* L*., commonly known as Java turmeric, is a popular dietary spice used for food in Asia, especially Indonesia and India. In addition, it has been used as a traditional medicinal plant to reduce the sensitivity of the liver to lipid peroxidation, as well as therapeutic properties against cancer, abnormally reduced fatty acid levels, and inflammatory disorders in adipose tissue [6–8]. TE has also been proven to be effective in NAFLD treatment. In modern research, growing evidence indicates that TE markedly affects liver diseases, such as acute liver injury, hepatic steatosis, and oxidative stress and inflammation in liver which is associated with NAFLD [9, 10]. However, compared with studies of its clinical application and pharmacological effect, there is inadequate detailed information on the mechanism involved in the therapeutic effects of TE.

As a crucial component of systems biology, metabolomics offers a noninvasive platform and holistic insight into the whole metabolic profile by detecting 1000s of molecules in various biological fluids, such as the urine, saliva, and blood [11]. By analyzing specific early biomarkers during disease or drug treatment, metabolomics has shown great potential in understanding disease mechanisms, identifying diagnostic biomarkers or drug targets, and the relationship between a substance and metabolic pathways. Moreover, metabolomics reflects the terminal symptoms of metabolic network of biological systems in holistic context [12]. This trait is well coincident with the integrity and systemic feature of TCM, indicating it is a comprehensive analytical approach to clarify the underlying efficacies and therapeutic mechanisms of TCM [13, 14].

In this study, metabolic profiling with UHPLC-Q-TOF-MS was performed to obtain a systematic view of the mechanism of TE as an effective treatment for NAFLD. In addition, to provide a deep understanding of the mechanism, specific biomarkers and unique biochemical pathways were applied, coupled with multivariate data analysis techniques. Our findings might also provide guidance in the improvement of TCM therapy strategies in the future.

## 2. Materials and Methods

### 2.1. Chemicals and Reagents

Acetonitrile (HPLC grade) and formic acid (HPLC grade) were obtained from Sigma-Aldrich (St. Louis, MO, USA). Distilled water was purified using a Milli-Q ultrapure water system (Millipore, Billerica, MA). All other reagents were of analytical grade. The total turmeric extract was an ethanol extract of the dried root of Curcuma longa* L* supplied by Shenwei pharmaceutical group (Hebei, China).

### 2.2. Animal Handling and Sample Preparation

#### 2.2.1. Animal Handling

Male Sprague-Dawley rats (200 ± 20 g) were supplied by the laboratory animal center of the Military Medical Science Academy of the PLA (permission number SCXK-(A) 2012-0004). The room temperature was regulated at 24 ± 2°C and a humidity of 50 ± 5%. The research was conducted in accordance with the NIH policy. All efforts were made to alleviate the suffering of animals. After acclimatization, animals were randomized into control group, model group (fed with HFD), positive control group (Compound Methionine and Choline Bitartrate Tablets [15, 16], 162 mg/kg), LD-TE group (50 mg/kg), MD-TE group (100 mg/kg), and HD-TE group (200 mg/kg), 10 rats per group. All rats were fed a HFD ad libitum (control group was fed a regular diet) for 8 weeks. The TE doses were given after rats developed NAFLD. Besides, four rats in each group were sacrificed randomly, the livers were collected, and pathological changes in the liver tissues were observed by H&E staining and oil O staining. Histological changes were assessed by a modification of the scoring system for grading and staging for NASH described by Brunt et al. [17]. The histological evaluation of the liver sections was performed blindly. Scoring of morphological changes was shown in Table 1 [Table tab2]. The drugs were dissolved in 0.5% sodium carboxymethyl cellulose solution and orally administered for 6 weeks after rats developed NAFLD. Rats in the control group were intragastrically administered an equivalent volume of solvent. The rats were fasted for 12 h before the experiments, but tap water was provided ad libitum.

#### 2.2.2. Sample Preparation

Animals were euthanized on the last day. Blood samples were collected and centrifuged at 3000 ×g for 10 min at 4°C. The supernatants were separated and stored at −80°C for metabolomics analysis. An Olympus AU5400 (Olympus, Tokyo, Japan) automated clinical biochemistry analyzer was employed to measure the serum ALT, AST, TC, TG, HDL-c, and LDL-c. Portions of liver tissues were excised, fixed in 4% paraformaldehyde solution, and stained with hematoxylin and eosin.

#### 2.2.3. Western Blotting

Liver tissue (0.1 g) was homogenized and subsequently lysed in ice-cold lysis buffer containing 1 mM phenylmethylsulfonyl fluoride and a protease inhibitor mixture. The sample was centrifuged at 8000 ×g and 4°C for 10 min to remove any debris. After centrifugation, the supernatant was aliquoted and stored at −80°C for the western blotting assay to detect PEMT, PSD, and PLA2G4. Fifty micrograms of total liver protein was separated by 12% SDS-polyacrylamide gel electrophoresis and transferred to a nitrocellulose membrane. Immunodetection was performed using rabbit anti-PEMT antibody (1 : 1000), anti-PSD antibody (1 : 1000), anti-PLA2G4 antibody (1 : 1000), and anti-*β* actin antibody (1 : 1000) in a solution of 5% milk in Tris-buffered saline and 0.05% Tween-20. After incubation with the appropriate secondary peroxidase-conjugated antibody, the membrane was washed in TBST for 60 min, and the immunoreactive bands were visualized with chemiluminescence.

### 2.3. Handling of Serum Samples

Six rats per group were analyzed. A total of 250 *μ*L thawed serum samples and 750 *μ*L prechilled acetonitrile were transferred to 1.5 mL polypropylene tubes, and the mixture was vortexed for 30 s and allowed to stand for 20 min at 4°C before use. The samples were centrifuged at 10,000 rpm for 10 min at 4°C, and the supernatant was transferred into new tubes.

### 2.4. Metabolic Profiling and Metabolite Analysis

#### 2.4.1. Chromatography and Mass Spectrometry

An Agilent 6550 UHPLC-Q-TOF/MS system was used for analysis. A ZORBAX RRHD 300 SB-C18 column (2.1 × 100 mm, 1.8 *μ*m, Agilent, USA) was performed to separate the serum constituents. Samples were maintained at 4°C and the column temperature was set at 30°C. The injection volume was set at 4 *μ*L. The mobile phases were comprised of 0.1% formic acid in acetonitrile (solvent A) and 0.1% formic acid in water (solvent B). The gradient was operated at a flow rate of 0.30 mL/min employing a linear gradient of 95% A at 0.0–1.0 min, 95–60% A at 1.0–9.0 min, 60–10% A at 9.0–19.0 min, 10–0% A at 19.0–20.0 min, and 0% A at 20.0–25.0 min. A QC sample mixed with 10 *μ*L in each sample was injected as a blank after every 5 samples were injected.

Electrospray capillary voltage was 3.0 kV in negative ionization mode and 4.0 kV in positive ionization mode. The gas temperature was 225°C in the ESI+ and 200°C in the ESI− mode. Gas flow was 11 L/min. Sheath gas temperature was 350°C and sheath gas flow was 12 L/min. Nozzle voltage was 500 V in both the negative and positive modes. The mass data were collected from *m*/*z* 80 to 1000 Da.

#### 2.4.2. Statistical Analysis

Sample data were extracted by MassHunter Profinder software (Agilent, California, USA). Data were presented as mean ± SD and scaled to Pareto variance. The SPSS 20.0 software program (IBM, SPSS, Chicago, IL, USA) was used for the statistical analysis. The statistical significance between the groups was analyzed by ANOVA followed by Tukey's post hoc test, with *P* < 0.05 set as the confidence level of statistical significance (highly significant at *P* < 0.01).

#### 2.4.3. Multivariate Data Analysis

The data were imported to SIMCA-P+ 13.0 software (Umetrics AB, Umea, Sweden) prior to modeling, and Pareto scaling and column centering were used for every variable. Then, multivariate statistical analyses, including PCA and PLS-DA were applied. These variables with a higher VIP value (VIP ≥ 1.0) and |*p*(corr)|≥0.58 in OPLS-DA were further evaluated. Only the variables (*P* < 0.05 in ANOVA and above 2-fold changes) were selected as candidates and subjected to further identification.

#### 2.4.4. Biomarkers Identification and Pathway Enrichment Analysis

Compounds with significant changes between groups (*P* value < 0.05 and fold-change > 2) were selected as biomarkers. The potential biomarkers were identified by Agilent MassHunter PCDL Manager software. 20 potential biomarkers and KEGG numbers were subjected to Metaboanalyst [18] (http://www.metaboanalyst.ca/) for further pathway enrichment analysis.

## 3. Results

### 3.1. Histopathological Observations and Biochemical Analysis

**Figure 1:**
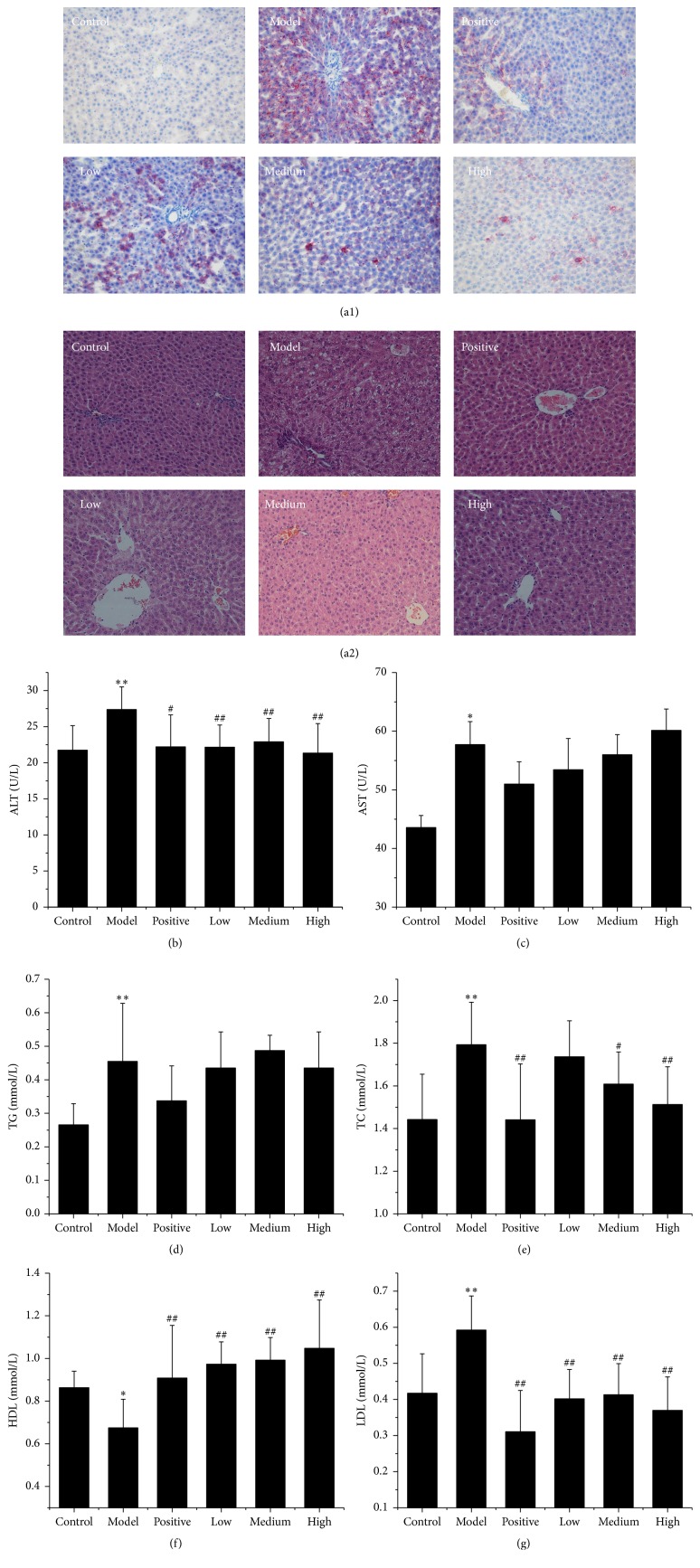
(a1) Representative light photomicrographs of rat liver specimens for oil red O staining (200x magnification); (a2) representative light photomicrographs of rat liver specimens for H&E analysis (200x magnification); (b–g) the serum levels of liver function indexes were assayed. (b) ALT; (c) AST; (d) TG; (e) TC; (f) HDL-c; (g) LDL-c. Data are expressed as the mean ± SD; control: control group; model: HFD group; positive: positive control group (CMCB); low: LD-TE; medium: MD-TE; high: HD-TE. ^*∗∗*^ *P* < 0.01, ^*∗*^ *P* < 0.05 compared with the control group; ^##^ *P* < 0.01, ^#^ *P* < 0.05 compared with the model group.

Histological evaluations provided visual evidence for the injury of HFD-induced NAFLD and the protective efficacy of TE on NAFLD. As demonstrated in Figure 1, oil red O staining showed that HFD rats developed severe macrosteatosis (Figure 1(a1)) and H&E staining showed an increase in ballooning degeneration and inflammation in the liver of HFD groups, whereas no such change was observed in the control group (Figure 1(a2)). These changes were more prominent in the model group than in other groups. The generally required histopathologic observations for NAFLD diagnosis are macrosteatosis, lobular inflammation, and hepatocyte ballooning. These three features were all observed in the HFD rats. After being treated with TE doses, macrosteatosis, lobular inflammation, and hepatocyte ballooning were ameliorated.

Morphological changes were assessed and the scoring of morphological changes was shown in Table 1. The livers of the control group had no signs of macrovesicular steatosis. Minimal microvesicular steatosis and minimal lobular and portal inflammatory changes were present. The livers of the HFD rats showed evidence of mild to moderate deposition of macrovesicular and microvesicular steatosis. After being treated with TE doses, macrovesicular and microvesicular steatosis and portal inflammation were significantly reduced in HD-TE and MD-TE livers.

To evaluate the therapeutic effects of TE, the levels of ALT, AST, TG, TC, HDL, and LDL in serum were compared with those of control groups. As shown in Figure 1(b), HFD-induced liver injuries in the model group rats were significantly different compared with the control group. The results showed that AST and TG were significantly elevated in HFD-induced liver injuries, but serum AST and TG levels showed no significant change in administered groups compared with the model group. However, the serum level of ALT was significantly reduced after being treated with HD, MD, and LD-TE groups, respectively. In addition, the other biochemical indices, including serum TC, HDL, and LDL, were decreased significantly in rats treated with 3 doses (Figures 1(b)–1(g)).

### 3.2. Multivariate Statistical Analysis and Potential Biomarkers

**Figure 2:**
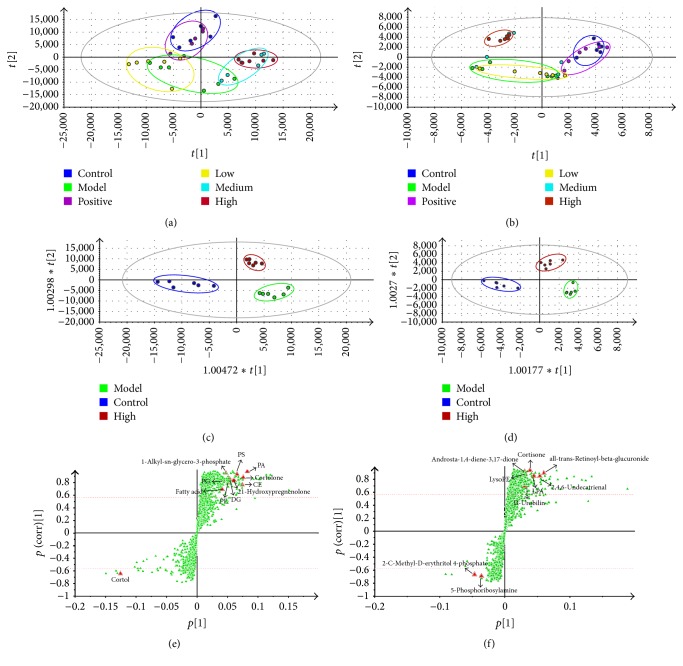
(a, b) PCA score plot of all groups in the ESI+ mode and ESI− mode. Blue circles: control group; green circles: model group (HFD group); violet circles: positive control group (CMCB); yellow circles: LD-TE group; cyan circles: MD-TE group; red circles: HD-TE group. (c, d) OPLS-DA score plot of the control group, model group, and HD-TE group in ESI+ mode and ESI− mode. (e, f) S-plot of the control group, model group, and HD-TE group in ESI+ mode and ESI− mode.

PCA was initially used as an unsupervised statistical method to study the metabolomic differences between control, model, positive control, and TE dose groups. A score plot provided a direct image of observational clusters. As shown in Figures 2(a) and 2(b), the clustering significantly differed between the control group, model group, and HD-TE group. However, the clustering did not distinguish the model group, LD-TE group, and MD-TE group well in PCA. The results of the PCA indicated that the model of NAFLD induced by HFD was successfully reproduced, and HD-TE had a better effect on NAFLD. Further multivariate statistical analysis was necessary to discern the relationship among control, model, and HD-TE groups.

Supervised OPLS-DA is a powerful method to pick out discriminating ions that are contributing to the classification of samples and remove noncorrelated variations contained within spectra. Thus, OPLS-DA was applied to investigate potential biomarkers between the control group, model group, and HD-TE group. As shown in OPLS-DA score plot (Figures 2(c) and 2(d)), there was a distinguished classification among the clustering of the normal, model, and HD-TE groups in the ESI+ mode and ESI− mode, suggesting that metabolic profiles significantly changed in 3 groups. Commonly, the R^2^Y and Q^2^Y provide an estimate of predictive ability in OPLS-DA model. In the ESI+ model, the parameters for classification from the software were R^2^Y = 0.937 and Q^2^Y = 0.819, and, in the ESI− model, R^2^Y = 0.930 and Q^2^Y = 0.722, respectively, indicating that the OPLS-DA model was well established. To further demonstrate that the responsibility of each ion is more intuitive in these variations, an S-plot was employed (Figures 2(e) and 2(f)). The more away a red triangle is from the origin, the more influence it would have on the separation of samples. Thus, the further metabolite ions from the origin exhibiting a higher VIP and |*p*(corr)| were potential biomarkers, which are responsible for the difference between 3 groups. In this work, VIP ≥ 1.0 and |*p*(corr)|≥0.58 were used as a screening standard to select potential metabolites. We marked the variables with red triangles according to their VIP value and |*p*(corr)| in ESI+ mode and ESI− mode.

### 3.3. Identification of Potential Metabolites

620 variables were selected as the candidates according to a threshold of VIP ≥ 1.0, and |*p*(corr)|≥0.58 in OPLS-DA among the 2000 peaks. Then, candidates that differed among the groups with a significant *P* value below 0.05 and a fold-change greater than 2 times were identified with the PCDL database. Finally, 20 potential biomarkers showed significant differences among the three groups. The corresponding retention time, *m*/*z*, formula of the biomarkers, and variation trends in the different groups are listed in Table 1. The detailed changes of potential biomarkers were also identified among the two respective groups.

### 3.4. Changes in Potential Metabolites in NAFLD with Different Doses of TE Treatment

**Figure 3:**
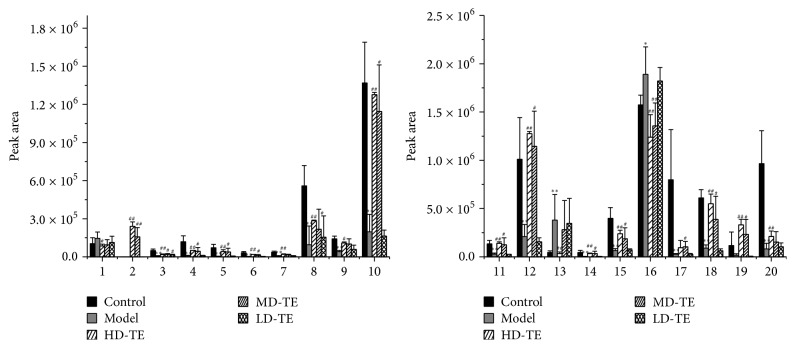
Changes in potential metabolites in NAFLD with different doses of TE treatment. Metabolites 1–20 were shown in Table 2. ^*∗∗*^ *P* < 0.01, ^*∗*^ *P* < 0.05 compared with the control group; ^##^ *P* < 0.01, ^#^ *P* < 0.05 compared with the model group.

To further evaluate the reversed condition of the potential biomarkers by administration of TE doses more intuitively, we analyzed changes in 20 potential metabolites. The relative peak intensities of the 20 metabolites identified in different groups were shown in Figure 3. Compared with the control group, metabolites 13 and 16 were both significantly upregulated; meanwhile the significantly downregulated 4, 5, 6, 7, 9, 14, 8, 10, 11, 12, 15, 17, 18, and 20 were both identified in NAFLD group. The significantly perturbed metabolite was most pronounced in response to the distinction between NAFLD and healthy states. Additionally, compared to the NAFLD group, biomarkers 1, 2, 4, 5, 7, 8, 9, 10, 11, 12, 13, 14, 15, 16, 17, 18, 19, and 20 were significantly reversed in the TE-treated group, and the other metabolites were also reversed to different degrees.

### 3.5. Western Blotting for Bile Acid Metabolism Confirmation

**Figure 4:**
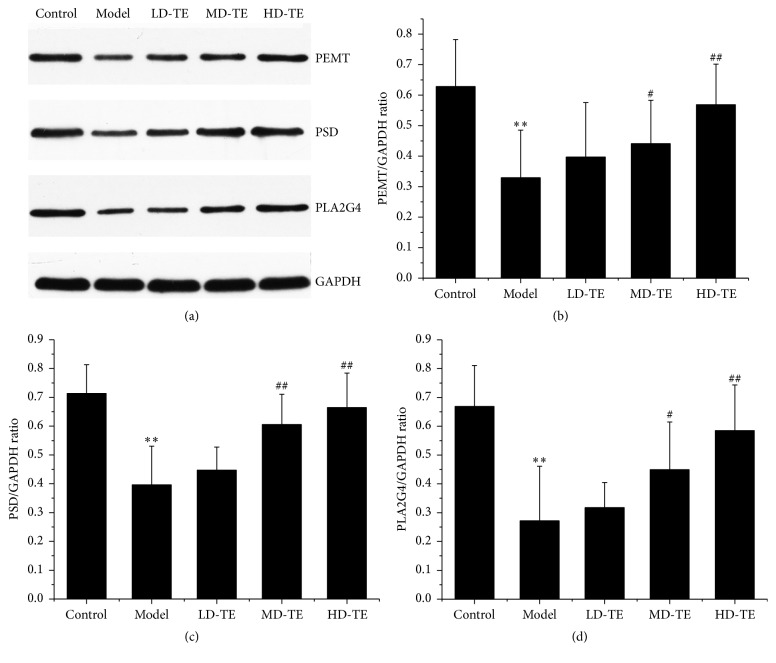
Western blotting for glycerophospholipid metabolism confirmation. (a) The western blot images of PEMT, PSD, and PLA2G4; (b) PEMT protein level in liver tissue; (c) PSD protein level in liver tissue; (d) PLA2G4 protein level in liver tissue. ^*∗∗*^ *P* < 0.01, compared with the control group; ^##^ *P* < 0.01, ^#^ *P* < 0.05 compared with the model group; control: control group; model: HFD group.

To ensure that TE primarily exerts its effect by regulating glycerophospholipid metabolism, we further explored the protein expressions of several zymoproteins such as PEMT, PSD, and PLA2G4. The results showed that the expression of PEMT, PSD, and PLA2G4 was markedly decreased in NAFLD rats compared with the control. Furthermore, treatment with HD-TE significantly increased the low expression levels of PEMT, PSD, and PLA2G4 in rats. However, these increases were limited in response to MD-TE and LD-TE (shown as in Figure 4).

## 4. Discussion

With the rapid growth of prevalence, NAFLD has become a common cause of chronic liver disease with a complex molecular pathogenesis. As reported previously, many pharmacological actions have been employed in NAFLD treatment [19, 20]. However, there are no widely accepted effective and safe medical therapy strategies for the treatment of NAFLD. Given this condition, the literature has reported the therapeutic effects of traditional Chinese medicine on NAFLD [4, 21, 22]. And* C. longa* was widely used for the treatment of NAFLD for many years [23].

**Figure 5:**
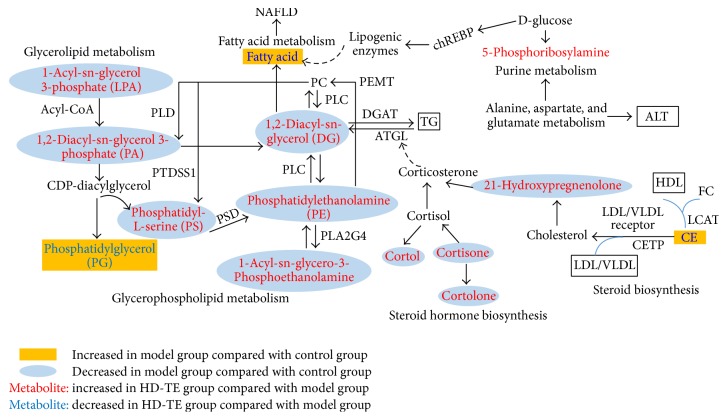
Schematic diagram of the disturbed metabolic pathway related to NAFLD and HD-TE treatment. The notations are as follows: yellow rectangle: metabolites increased in model group compared with control group; blue ovals: metabolites decreased in model group compared with control group; red metabolites: increased in HD-TE group compared with model group; and blue metabolites: decreased in HD-TE compared with model group; PEMT: phosphatidylethanolamine N-methyltransferase; PSD: phosphatidylserine decarboxylase; PLA2G4: phospholipase A2G4; ATGL: adipose triglyceride lipase; LCAT: lecithin cholesterol acyl transferase; CETP: cholesteryl ester transfer protein; PLC: phospholipase C.

In this study, the NAFLD model in rats was successfully reproduced, and TE was performed to treat NAFLD. Further, metabolomics and multivariate statistical analysis were used to analyze serum samples. Here, potential biomarkers associated with NAFLD were further analyzed, and the networks correlated with the potential biomarkers, the main disturbed metabolic pathway related to NAFLD, and the possible metabolic mechanisms of HD-TE treatment are summarized in Figure 5.

As demonstrated in Figure 5, we found that most pathways of the potential biomarkers were related to glycerophospholipid metabolism, glycerolipid metabolism, and steroid hormone biosynthesis according to the KEGG pathway maps (01100, metabolic pathways). Several lipid mediators typically associated with lipotoxicity such as diacylglycerols (DGs), oxysterols, and fatty acids are commonly found in metabolic syndrome, insulin resistance, and type 2 diabetes [24], which are frequent comorbidities associated with NAFLD [25].

Fatty acids are common lipid mediators produced by fatty acid synthases from acetyl-CoA and malonyl-CoA precursors, by lipases in the degradation of glycerolipids, or by phospholipase in the breakdown of glycerophospholipids [26]. NAFLD results from imbalanced lipid homeostasis in the liver, which may be induced by HFD. In agreement with this, the elevation of fatty acid was detected in the serum. Despite the fundamental physiological importance, an oversupply of FAs is highly detrimental, leading to impaired membrane function, mitochondrial dysfunction, inflammation, and cell death [27]. In this study, the increased fatty acid provided the evidence of decreased DG and glycerophospholipids.

In this study, 6 biomarkers were related to glycerophospholipid metabolism, and 3 biomarkers were associated with glycerolipid metabolism. The levels of LPA, PA, PE, LysoPE, PS, and DG were elevated, indicating the glycerophospholipid and glycerolipid metabolism were inhibited. LPA, a lysophospholipid, the second product of reactions catalyzed by phospholipase A2, is rapidly acylated with acyl-CoA, resulting in the maintenance of the normal and essential neural membrane glycerophospholipid composition. However, under pathological situations (ischemia), the overstimulation of phospholipase A2 results in a rapid generation and accumulation of free fatty acids, resulting in inflammation, oxidative stress, and neurodegeneration [28]. It was reported that a 20% reduction in the hepatic PA content and 35% reduction in the PA/LPA ratio was observed in NAFLD rats with expression of PNPLA3, which were associated with a 50% reduction in hepatic DG content and increased hepatic insulin sensitivity [29]. In addition, all mammalian cell types synthesized hepatocytes PC is synthesized by the sequential methylation of PE, a reaction catalyzed by the enzyme PEMT. The conversion of PE to PC catalyzed by PEMT was impaired, which corroborates previous findings showing that the deficiency of the PEMT pathway led to hepatic TG accumulation [30]. Accordingly, the inhibition of glycerophospholipid and glycerolipid metabolism were closely associated with NAFLD. In HD-TE group, the downregulated PG and upregulated DG, PE, LysoPE, PS, and LPA were detected. As a result, concentration of FAs and TG was decreased. Therefore, HD-TE markedly regulated the levels of these bioactive lipid molecules in the serum, which suggested that the observed prevention mechanism is closely related to elevated lipid metabolism and fatty acid metabolism.

Glucocorticoids and hypercortisolemia caused elevation of circulating FFAs. The basis of this phenomenon had been linked to the effect of glucocorticoids enhancing the adipose lipolysis response to various hormones [31]. A recent study revealed that physiological hypercortisolemia may contribute to potent stimulus of lipolysis. In this study, the levels of several glucocorticoids and hypercortisolemia, such as cortisone, cortol, cortolone, and 21-hydroxypregnenolone, were significantly decreased in NAFLD rats, indicating adipose lipolysis was inhibited. Notably, the metabolites were relevant to corticosterone, which had direct effects on ATGL, supporting the notion that glucocorticoids increase lipolysis through glucocorticoid-induced increases of lipase expression in response to treatment with HD-TE compared with NAFLD [32]. Finally, the lipolytic action of glucocorticoids liberates excess TG efflux from adipocytes to the bloodstream in HD-TE rats.

Abnormal cholesterol metabolism is closely related to HDL and LDL levels. The main atheroprotective mechanism of HDL is reverse cholesterol transport, whereby excess cholesterol is transported from peripheral tissues to the liver for elimination [33]. In contrast, cholesterol is removed from the liver to surrounding tissues by LDL, and LDL levels are related to the risk of atherosclerosis [34]. As shown in this network, HDL converts FC to CE by the LCAT enzyme. In humans, CE would be transferred to other lipoproteins by CETP, promoting the removal of CE from HDL in exchange for TG derived primarily from VLDL. Finally, it is absorbed in the liver by the VLDL/LDL receptor. Therefore, the downregulated level of CE indicates the regulation of abnormal cholesterol metabolism, contributing to regulated levels of HDL and LDL.

Lipid metabolism is regulated by multiple signaling pathways and generates a variety of bioactive lipid molecules, such as fatty acid, eicosanoids, DGs, PA, LPA, sphingosine, phosphatidylinositol-3 phosphate, and cholesterol, which are involved in the activation or regulation of different signaling pathways [27]. In this study, glycerophospholipid metabolism, glycerolipid metabolism, and steroid hormone biosynthesis were recognized as the main signaling pathways in NAFLD and TE treatment. Impaired glycerophospholipid metabolism and glycerolipid metabolism lead to the lipidosis in the liver, causing hepatocyte steatosis and inflammation. The changed metabolites, such as LPA, PA, PS, PE, DG, PG, and LysoPE, constitute both the phenotype and cause of the progression of NAFLD. In our study, several proteins in hepatocytes are also specifically changed during NAFLD, including PEMT, PSD, and PLA2G4. PEMT catalyzes PC synthesis from PE. Studies have shown that the deficiency of either of the PEMT pathways led to hepatic TG accumulation. PSD and PLA2G4 are the enzymes that catalyze PS and PE metabolism. Our data indicated that the expressions of PEMT, PSD, and PLA2G4 were decreased in NAFLD rats. The lower expression of enzymes could cause accumulation of lipids. Treatment with HD-TE was able to significantly increase the lower expression of PEMT, PSD, and PLA2G4 as well as bile acid such as LPA, PA, PS, PE, DG, PG, and LysoPE, indicating that the regulation of lipid metabolism effect of HD-TE on NAFLD was probably associated with the glycerophospholipid metabolism and glycerolipid metabolism signaling pathways. Furthermore, the in vitro effects of cortisol and cortisone on basal and stimulated lipolysis in human adipose tissue were studied in previous studies [35, 36]. Several glucocorticoids and hypercortisolemia, such as cortisone, cortol, cortolone, and 21-hydroxypregnenolone were detected in our study, and they may ultimately regulate lipolysis associated with the steroid hormone biosynthesis. In addition, the degradation of TG to DG was catalyzed by ATGL [37, 38], which catalyzed the initial step of lipolysis. As shown in schematic diagram, ATGL was affected by the upregulated 21-hydroxypregnenolone in the HD-TE group, and it was activated by downregulated CE associated with steroid biosynthesis. Therefore, the lipolytic effect of HD-TE was also probably relative to the steroid biosynthesis signaling pathways. Thus TE had powerful combined effects of regulating lipid metabolism by affecting glycerophospholipid metabolism, glycerolipid metabolism, and steroid hormone biosynthesis signaling pathways.

In this study, a UHPLC-Q-TOF-MS-based metabolomics approach was applied to investigate the biomarkers of NAFLD with the administration of TE. According to the metabolic pathways of these significantly changed metabolites, it is confirmed that TE had powerful combined effects of regulating lipid metabolism by affecting glycerophospholipid metabolism, glycerolipid metabolism, and steroid hormone biosynthesis signaling pathways. Moreover, the effects of glucocorticoids on adipose tissue metabolism are conflicting, and the contradictory effects of glucocorticoids on lipid metabolism occur through a number of different mechanisms, some of which are well defined and others remain to be elucidated. Our study can be applied in glucocorticoids on lipid metabolism research in the future.

## 5. Conclusion

A serum metabolomic study based on UHPLC-Q-TOF-MS was successfully performed to explore potential biomarkers in NAFLD and investigate the mechanism of TE treatment on NAFLD. With the help of serum biochemistry and histopathology, the HFD-induced NAFLD was confirmed. The control, model, and HD-TE groups were successfully discriminated by PCA and OPLS-DA. As a result, 20 significantly changed metabolites were identified as potential biomarkers associated with HD-TE of NAFLD. All the data yielded the conclusion that TE could reverse the pathological process of NAFLD through the powerful combined effects of regulating lipid metabolism by affecting glycerophospholipid metabolism, glycerolipid metabolism, and steroid hormone biosynthesis signaling pathways. The results implied that a UHPLC-Q-TOF-MS-based metabolomics approach could provide a systematic view of the characteristics of NAFLD. It also offered better guidance for the investigation of safe and effective herbal medicines for the prevention or treatment of NAFLD.

## Supplementary Material

The identification for the phytochemicals of the extract of total turmeric using LC-QTOF/MS, and UPLC-MS BPCs of samples from control, model, positive control and TE dose groups were contained in the Supplementary Material. The results of 11 pathways and the most affected pathways were also obtained.

## Figures and Tables

**Table 1:** Scoring of morphological changes.

	*n*	Macrovesicular steatosis	Microvesicular steatosis	Lobular inflammation	Portal inflammation
Control	6	0.0 ± 0.0	0.17 ± 0.41	0.33 ± 0.52	0.17 ± 0.41
Model (HFD)	6	1.67 ± 0.82^*∗∗*^	2.17 ± 0.75^*∗∗*^	1.33 ± 0.52^*∗∗*^	1.17 ± 0.41^*∗∗*^
Positive (CMCB)	6	0.33 ± 0.52^##^	1.00 ± 0.63^#^	0.67 ± 0.52^#^	0.33 ± 0.52^#^
LD-TE	6	0.83 ± 0.75	1.33 ± 0.82	1.33 ± 0.82	0.67 ± 0.52
MD-TE	6	0.33 ± 0.52^##^	1.17 ± 0.75^#^	0.83 ± 0.41	0.50 ± 0.55^#^
HD-TE	6	0.17 ± 0.41^##^	0.67 ± 0.52^##^	0.50 ± 0.55^#^	0.33 ± 0.52^#^

^*∗∗*^ *P* < 0.01 compared with the control group; ^##^ *P* < 0.01, ^#^ *P *< 0.05 compared with the model group.

**Table 2 tab2:** List and change trends of differential metabolites.

Number	RT (min)	Mass (*m/z*)	Metabolites	Formula	Model trend^a^	HD-TE trend^b^
1	0.95	206.0419	2-C-Methyl-D-erythritol 4-phosphate	C_5_H_13_O_7_P	↑	↓^#^
2	5.12	229.0371	5-Phosphoribosylamine	C_5_H_12_NO_7_P	—	↑^##^
3	14.46	360.1873	Cortisone	C_20_H_28_O_5_	↓^*∗∗*^	↑^##^
4	15.24	284.1776	Androsta-1,4-diene-3,17-dione	C_19_H_24_O_2_	↓^*∗∗*^	↑^##^
5	15.95	476.2441	all-trans-Retinoyl-beta-glucuronide	C_26_H_36_O_8_	↓^*∗∗*^	↑^##^
6	16.17	535.3668	1-Acyl-sn-glycero-3-phosphoethanolamine (LysoPE)	C_27_H_54_NO_7_P	↓^*∗∗*^	↑
7	16.34	434.2463	1-Acyl-sn-glycerol-3-phosphate [LPA(18:2(9Z,12Z)/0:0)]	C_20_H_39_O_7_P	↓^*∗∗*^	↑^##^
8	17.19	757.5842	Phosphatidylethanolamine [PE(15:0/22:2(13Z,16Z))]	C_42_H_80_NO_8_P	↓^*∗∗*^	↑^#^
9	17.20	164.1220	2,4,6-Undecatrienal	C_11_H_16_O	↓^*∗∗*^	↑^##^
10	17.42	368.2563	Cortol	C_20_H_36_O_5_	↓^*∗∗*^	↑^##^
11	17.45	332.2351	21-Hydroxypregnenolone	C_20_H_32_O_3_	↓^*∗∗*^	↑^##^
12	17.46	366.2473	Cortolone	C_20_H_34_O_5_	↓^*∗∗*^	↑^##^
13	18.49	408.3038	4Z,7Z,10Z,13Z,16Z,19Z,22Z,25Z-Octacosaoctaenoic acid (fatty acid)	C_28_H_40_O_2_	↑^*∗∗*^	↓^##^
14	19.31	588.2944	D-Urobilin	C_33_H_40_N_4_O_6_	↓^*∗∗*^	↑^##^
15	20.48	424.3008	1-Alkyl-sn-glycero-3-phosphate (1-octadecyl lysophosphatidic acid)	C_20_H_45_O_6_P	↓^*∗∗*^	↑^##^
16	22.63	428.3684	Cholesteryl ester (CE(12:0))	C_29_H_48_O_2_	↑^*∗*^	↓^##^
17	22.74	686.4913	1,2-Diacyl-sn-glycerol	C_45_H_66_O_5_	↓^*∗∗*^	↑^#^
18	22.76	720.4941	Phosphatidylglycerol [PG(14:0/18:1(9Z))]	C_38_H_73_O_10_P	↑^*∗*^	↓^##^
19	22.93	727.4572	Phosphatidyl-L-serine [PS(14:1(9Z)/18:3(9Z,12Z,15Z))]	C_38_H_66_NO_10_P	↓	↑^##^
20	23.04	660.5034	1,2-Diacyl-sn-glycerol 3-phosphate PA(P-20:0/14:0)	C_37_H_73_O_7_P	↓^*∗∗*^	↑^##^

^a^Compared to the normal group. ^b^Compared to the model group. The significantly changed biomarkers were flagged with (↓) downregulated, (↑) upregulated, and (—) invalid data; ^*∗*, #^ *P* < 0.05, ^*∗∗*, ##^ *P* < 0.01.

## Abbreviations

NAFLD: Nonalcoholic fatty liver disease
TE: Total turmeric extract
HFD: High-fat diet
CMCB: Compound Methionine and Choline Bitartrate Tablets
TCM: Traditional Chinese medicine
HD-TE: High dose of total turmeric extract
MD-TE: Medium dose of total turmeric extract
LD-TE: Low dose of total turmeric extract
ALT: Alanine amino transferase
AST: Aspartate amino transferase
TG: Triglyceride
TC: Total cholesterol
HDL-c: High-density lipoprotein cholesterol
LDL-c: Low-density lipoprotein cholesterol
PCA: Principal component analysis
OPLS-DA: Orthogonal partial least squares
PA: 1,2-Diacyl-sn-glycerol 3-phosphate
PG: Phosphatidylglycerol
PS: Phosphatidyl-L-serine
PE: Phosphatidylethanolamine
PC: Phosphatidylcholine
CE: Cholesteryl ester
DG: Diglyceride
FC: Free cholesterol
LCAT: Lecithin cholesterol acyltransferase enzyme
CETP: CE transfer protein
VLDL: Very low-density lipoprotein
PEMT: Phosphatidylethanolamine N-methyltransferase
PSD: Phosphatidylserine decarboxylase
PLA2G4: Phospholipase A2G4
ATGL: Adipose triglyceride lipase
LCAT: Lecithin cholesterol acyl transferase
CETP: Cholesteryl ester transfer protein
PLC: Phospholipase C.
