# Supplementary material for: Untargeted Metabolomics Reveals Intervention Effects of Total Turmeric Extract in a Rat Model of Nonalcoholic Fatty Liver Disease
Source: Evid Based Complement Alternat Med. 2016 May 30;2016:8495953. doi: 10.1155/2016/8495953 (PMC4904104; doi:10.1155/2016/8495953)
Supplement: Supplementary file 1 — The identification for the phytochemicals of the extract of total turmeric using LC-QTOF/MS, and UPLC-MS BPCs of samples from control, model, positive control and TE dose groups were contained in the Supplementary Material. The results of 11 pathways and the most affected pathways were also obtained. [file 8495953.f1.pdf]

Untargeted metabolomics reveals intervention effects of total turmeric extract in a rat model of nonalcoholic fatty liver disease

Ya Wang[1, 2‡], Ming Niu[2‡], Ge-liu-chang Jia[2‡], Rui-sheng Li[2], Ya-ming Zhang[2], Cong-en Zhang[2], Ya-kun Meng[2], He-rong Cui[2], Zhi-jie Ma[3], Dong-hui Li[1*], Jia-bo Wang[2*], Xiao-he Xiao[2*]

[1] *Pharmacy College, Jinzhou Medical University, Jinzhou 121000, PR China.*

[2] *China Military Institute of Chinese Medicine, 302 Military Hospital, Beijing, PR China.*

[3] *Beijing Friendship Hospital, Capital Medical University, Beijing, PR China.*

[1] ‡ These authors contributed equally to this work.

* To whom correspondence should be addressed: Dong-hui Li, Pharmacy College, Jinzhou Medical University, E-mail address: lidonghuilx@sina.com, Tel/Fax: +86 0416 4673252; Jia-bo Wang, China Military Institute of Chinese Medicine, 302 Military Hospital, E-mail address: pharm_sci@126.co*m*, Tel/Fax: +86 10 6693 3325; Xiao-he Xiao, Integrative Medical Center, 302 Military Hospital, E-mail address: pharmacy302xxh@126.com, Tel/Fax: +86 10 66933 322.

LC-QTOF/MS identification for the phytochemicals of the extract of total turmeric

For qualitative analysis, chromatographic analysis was performed using an Agilent 1290 UHPLC system. The analysis was conducted on an Agilent Poroshell 120 EC-C18 column (2.1 × 100 mm, 2.7 μm). The mobile phases used were solvent A (H_2O), solvent B (acetonitrile), with gradient elution as follows: 5% – 40% B at 0 – 9 min, 40% – 95% B at 9 – 12 min, 95 % B at 12 – 22 min. The flow rate was kept at 0.3 mL/min, followed by column re-equilibration for 5 min. The column and autosampler were maintained at 25ºC and 4°C, respectively. The injection volume of reference compounds and samples was 1 μL.

Detection was performed with the 6550 UHPLC/QTOF/MS instrument equipped with an ESI instrument, using full-scan mode with the mass range set at m/z 50–1200 in positive ion mode. The ESI conditions involved nitrogen drying gas at a flow rate of 11 L/min and a temperature of 225°C, a nebulizer gas (nitrogen) pressure of 45 psig, a capillary voltage of 4000 V, a fragmentor voltage of 230 V, and an octopole RF voltage of 750 V. Data acquisition and processing were conducted with the MassHunter Qualitative analysis software. Figure S1 A shows UHPLC-UV (420 nm) chromatograms of total turmeric extract; Figure S1 B shows a visual examination of MS BPC chromatograms. The details of identified compounds are summarized in Table S1. We applied the 3 collision energies of 10, 20 and 40 eV to produce the main product ion at m/z 369.1329 of curcumin. The main product ion at collision energies of 10 eV is shown in Fig. S2.

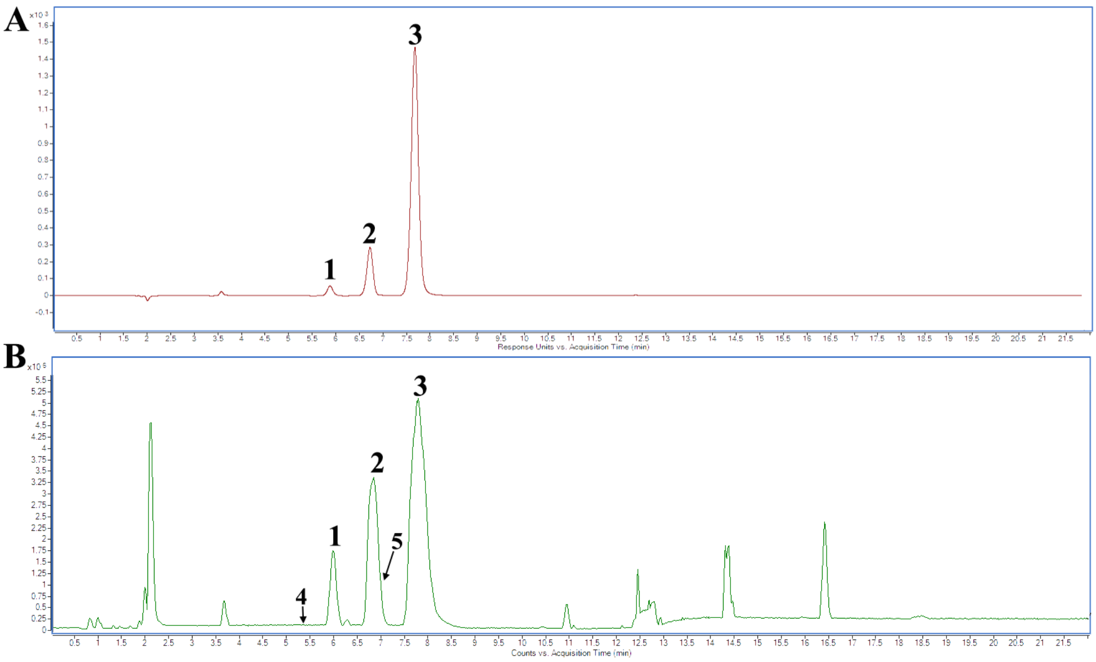

Fig. S1. (A) UHPLC-UV (420 nm) chromatograms of total turmeric extract. 1, bisdemethoxycurcumin; 2, demethoxycurcumin; 3, curcumin. (B) Typical BPC chromatograms of total turmeric extract in ESI+ mode. 1, bisdemethoxycurcumin; 2, demethoxycurcumin; 3, curcumin; 4, curcumol；5, dehydrodeguelin. Compound 4 and 5 were found using MS1 extracted ion chromatograms.

Table S1 Identification of chemical constituents in turmeric extract

NO.	R T (min)	Identification	Formula	$[M + H]^+$ m/z	$[M + Na]^+$ m/z	Error (ppm)	Product ions (m/z)
1	5.968	bisdemethoxycurcumin	$C_{19}H_{16}O_4$	309.1127	331.0946	-1.83	147.0435, 225.0904
2	6.932	demethoxycurcumin	$C_{20}H_{18}O_5$	339.1232	361.0974	-1.49	147.0436, 255.1010
3	7.780	curcumin	$C_{21}H_{20}O_6$	369.1339	391.1158	-1.45	177.0547, 285.1119
4	5.253	curcumol	$C_{15}H_{24}O_2$	—	259.1675	-2.51	219.0841
5	6.999	dehydrodeguelin	$C_{23}H_{20}O_6$	393.1315	415.1137	-1.36	393.1315

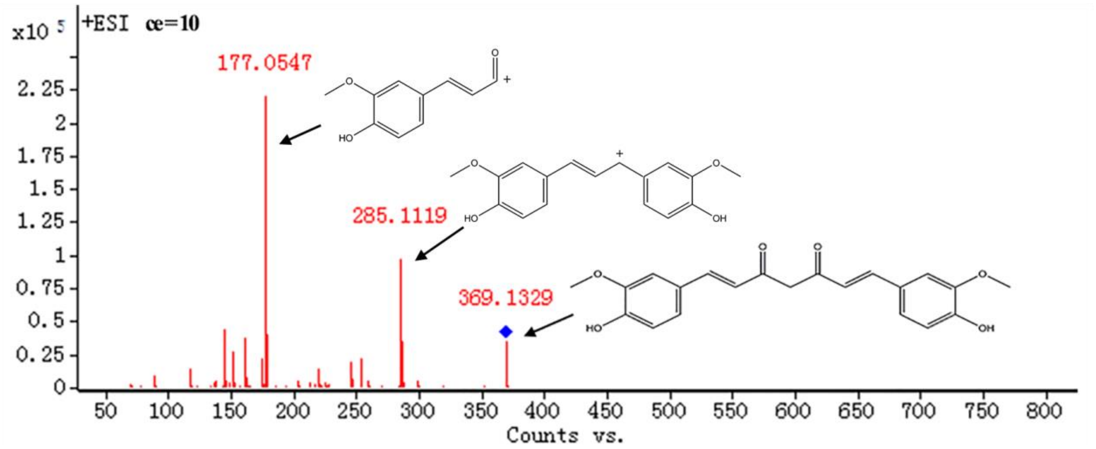

Fig. S2. Product ion scan spectra of curcumin to monitor the fragmentation transitions of m/z 369.1329 → m/z 177.0547 in ESI+ mode.

The typical base peak chromatograms (BPC) of samples derived from control, model, positive control and TE dose groups in ESI+ mode are presented in Fig. S3 by using the optimal LC-MS conditions described above. Low molecular mass metabolites could be separated well in a short time.

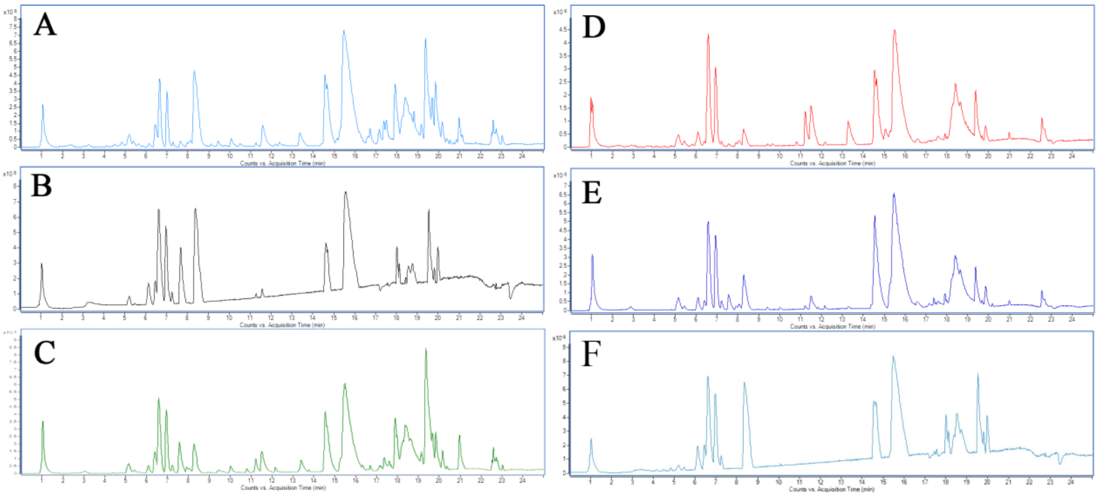

Fig. S3**.** UPLC-MS BPCs of samples from each group. A: control group; B: model group; C: positive control group; D: low dose group; E: middle dose group; F: high dose group.

Metaboanalyst 3.0 software was used for further pathway enrichment analysis of above biomarkers. 11 pathways obtained show in Table S2. Among these, the most affected pathways are shown in Fig. S4.

Table S2 Result from Pathway Analysis with metaboanalyst 3.0

Pathway name	Total	Hits	-Log(p)	Raw p	Impact
Glycerophospholipid metabolism	30	6	12.608	3.35E-06	0.2423
Glycerolipid metabolism	18	3	6.0525	0.0024	0.1364
Fatty acid metabolism	39	2	2.0965	0.1229	0.0027
Ether lipid metabolism	13	1	1.6789	0.1866	0
Glycosylphosphatidylinositol(GPI)-anchor biosynthesis	14	1	1.6121	0.1995	0.0439
Retinol metabolism	17	1	1.4399	0.2370	0
Steroid hormone biosynthesis	70	2	1.1993	0.3014	0.0169
Alanine, aspartate and glutamate metabolism	24	1	1.1456	0.3180	0
Inositol phosphate metabolism	26	1	1.0799	0.3396	0
Steroid biosynthesis	35	1	0.8462	0.4291	0
Purine metabolism	68	1	0.4036	0.6679	0.0276

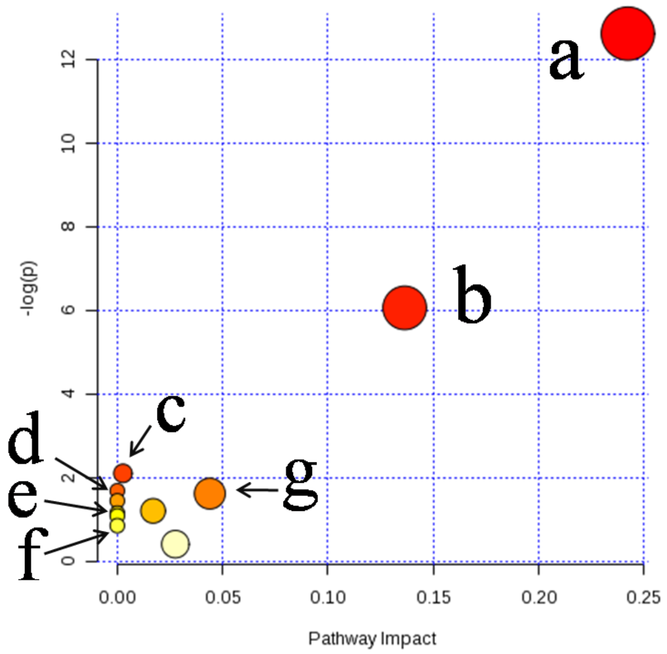

Fig. S4. Summary of pathway analysis with Metaboanalyst 3.0. a, Glycerophospholipid metabolism; b, Glycerolipid metabolism; c, Fatty acid metabolism; d, Ether lipid metabolism; e, Alanine, aspartate and glutamate metabolism; f, Steroid biosynthesis; g, Glycosylphosphatidylinositol (GPI)-anchor biosynthesis.
